# The relationship between bite force, morphology, and diet in southern African agamids

**DOI:** 10.1186/s12862-021-01859-w

**Published:** 2021-06-21

**Authors:** W. C. Tan, J. Measey, B. Vanhooydonck, A. Herrel

**Affiliations:** 1 Herpetology Section, Zoologisches Forschungsmuseum Alexander Koenig, Adenauerallee 160, 53113 Bonn, Germany; 2 Institut für Zoologie, Rheinische Friedrich-Wilhelms-Universität Bonn, Poppelsdorfer Schloss, Bonn, Germany; 3 Laboratoire EBI Ecologie and Biologie des Interactions, UMR CNRS 7267, Université de Poitiers, UFR Sciences Fondamentales et Appliquées, Poitiers, France; 4 Centre for Invasion Biology, Department of Botany and Zoology, Stellenbosch University, Stellenbosch, South Africa; 5 Department of Biology, University of Antwerp, Universiteitsplein 1, 2610 Antwerpen, Belgium; 6 Département Adaptations du Vivant, UMR 7179 C.N.R.S/M.N.H.N., Bâtiment d’Anatomie Comparée, 55 rue Buffon, 75005 Paris, France

**Keywords:** Agama, Diet, Habitat, Lizard, Morphometrics, Performance

## Abstract

**Background:**

Many animals display morphological and behavioural adaptations to the habitats in which they live and the resources they exploit. Bite force is an important whole-organism performance trait that allows an increase in dietary breadth, the inclusion of novel prey in the diet, territory and predatory defence, and is important during mating in many lizards.

**Methods:**

Here, we study six species of southern African agamid lizards from three habitat types (ground-dwelling, rock-dwelling, and arboreal) to investigate whether habitat use constrains head morphology and bite performance. We further tested whether bite force and head morphology evolve as adaptations to diet by analysing a subset of these species for which diet data were available.

**Results:**

Overall, both jaw length and its out-lever are excellent predictors of bite performance across all six species. Rock-dwelling species have a flatter head relative to their size than other species, possibly as an adaptation for crevice use. However, even when correcting for jaw length and jaw out-lever length, rock-dwelling species bite harder than ground-dwelling species. Diet analyses demonstrate that body and head size are not directly related to diet, although greater in-levers for jaw closing (positively related to bite force) are associated to an increase of hard prey in the diet. Ground-dwelling species consume more ants than other species.

**Conclusions:**

Our results illustrate the role of head morphology in driving bite force and demonstrate how habitat use impacts head morphology but not bite force in these agamids. Although diet is associated with variation in head morphology it is only partially responsible for the observed differences in morphology and performance.

**Supplementary Information:**

The online version contains supplementary material available at 10.1186/s12862-021-01859-w.

## Background

The adaptive significance of phenotypic traits has been of interest to evolutionary biologists for centuries [1]. Phenotypic variation is shaped by evolutionary and ecological processes with traits promoting survival and reproduction ultimately being selected. Therefore, understanding the relationship between morphology and function and how it is related to ecology is crucial to understand the evolution of phenotypic diversity [2, 3]. Performance traits related to locomotion and feeding are the most common functional traits tested as they are likely targets of selection given their role in survival and reproduction.

In lizards, variation in head morphology is relevant in many ecological (feeding, habitat and refuge use) and social contexts (territorial display, mating, and aggressive interactions). To test the possible adaptive nature of variation in head morphology, many studies have measured bite force. This performance trait has been related to both diet and territory defence [4–8]. The relationship between head shape and bite force is thought to be rather straightforward as the jaws can be approximated by a simple lever system. Moreover, larger heads (length, width and height) should result in an increase in bite force as they provide more space for the jaw adductor muscles [4, 6]. These inferences are supported by biomechanical models [9, 10] which show that taller and wider heads can accommodate more jaw adductor muscles that are pennate and thus can provide more force for a given volume [6, 11]. An increase in lower jaw length increases the out-lever of the jaw system which should reduce bite force as the lower jaw act as a lever system which transmits the input force from muscles to the out-lever arm to produce an output force [10].

However, bite force is not the only aspect influencing head shape. Ecological constraints such as habitat use may be important factors driving the evolution of head shape. For instance, animals living within rocky habitats hide in cervices when escaping from predators [11, 12]. To be able to do so they likely benefit from flat heads and bodies. However, given the importance of head height in driving bite force, a trade-off may exist between bite force and the ability to use narrow crevices. The importance of habit use in driving the evolution of the cranial morphology has been documented previously for some lizards [12–15]. Habitat use may also impact the availability of food resources, which may in turn drive the evolution of morphology and bite performance [16]. Lizards with larger heads can be expected to consume larger and harder prey due to the gape and bite force advantage conferred by these traits [5–7, 17, 18]. In addition to the above cited environmental constraints that are likely to affect bite force, bite force may also evolve through sexual selection. For example, in species where males engage in fights to defend territories or compete for access to mates, high bite forces likely result in increased fitness [19, 20].

Agamids are widespread across the African continent. They present a particularly interesting group to study ecomorphological relationships due to the shared evolutionary history and geographical distribution [21]. They are thought to have undergone rapid diversification, radiating into multiple clades about 10 Mya [21]. A recent study has shown divergence in limb morphology and locomotor performance between southern African agamas utilising different habitats, suggesting ecological differentiation between these species [22]. Since habitat is likely an important factor shaping limb and locomotor variation in these agamas, we expect this to drive variation in cranial morphology, bite force, and possibly even diet. However, the ecomorphological relationships between habitat use, morphology, performance and diet remain poorly known in agamids (but see [22, 23]). These lizards generally adopt a sit-and-wait foraging strategy, feeding predominantly on active prey such as ants, beetles and flying insects [24, 25] which may impact how prey availability drives variation in head morphology and bite force.

In this study, we studied six species of agamas from contrasting habitat types, representing rock-dwelling (*Agama atra, A. anchietae* and *A. aculeata distanti*), ground-dwelling (*A. aculeata aculeata* and *A. armata*) and arboreal (*Acanthocercus atricollis*) habitats [22, 26]. The genus *Agama* only consists of species living mostly in terrestrial and rocky habitats. Their closest extant group—the genus *Acanthocercus* however, contains 13 species of arboreal and rock-dwelling lizards [27]. Therefore, even though belonging to a different genus, the species *Acanthocercus atricollis* is the only agamid species found in southern Africa with an arboreal lifestyle [26]. Although occupying very different habitats, *A. a. aculeata* and *A. a. distanti* are two subspecies of *A. aculeata* belonging to a species complex which has not been completely resolved. We first explored the relationship between head morphology, bite force, and diet. We expect longer, wider, and taller heads and larger jaw closing in-levers to be associated with an increase in bite force [4, 11]. We further compared the association between head morphology and bite force in species from different habitats. Because rock-dwelling species possess flatter heads, allowing them to hide in rock crevices [11, 28], we expect reduced bite forces in these species. We hypothesised ground-dwelling species to have taller heads and thus higher bite forces [13, 14]. Finally, we predict species with larger heads and higher bite forces to include larger and harder prey into their diet [7, 17]. To test these hypotheses, we compared our findings with published data on stomach contents from four of the six examined agama species [24].

## Results

A multiple regression performed on the head measures with bite force as the dependent variable retained a single significant model (*R*^2^ = 0.94; *P* < 0.01) with the jaw out-lever (β = 0.52) and lower jaw length (β = 0.45) as significant predictors (Table 1). Most head variables (head length and width, lower jaw, jaw out-lever and snout length) were, however, highly and positively correlated with bite force (Table 1).

**Table 1:** Correlation analysis and stepwise multiple regression analyses with bite force as the dependent variable and head morphological traits as independent variables. All variables were log_10_ transformed

	*Pearson correlation analysis*		*Stepwise regression analysis*^*1*^	
	*r*	P	β	P
Bite force				
Head length	0.94	< 0.01		
Head width	0.92	< 0.01		
Head height	0.84	0.05		
Lower jaw length	0.96	< 0.01	0.45	< 0.01
Jaw out-lever	0.96	< 0.01	0.52	< 0.01
Snout length	0.90	< 0.01		
In-lever for jaw opening	0.54	0.74		
In-lever for jaw closing	0.48	0.89		

*r* Pearson correlation coefficient, β regression coefficient, P P-value

^*1*^Adjusted *R*^2^ (the coefficient of determination) = 0.94

### Morphology, performance and habitat association

Habitat groups differed significantly in body size (*F*_2,144_ = 24.94, *P* < 0.01). Post-hoc tests revealed that arboreal species were significantly larger than other groups, followed by rock-dwelling species (Table 2). The multivariate analyses of covariance (MANCOVA) with SVL as covariate showed significant differences in head shape between habitat groups (Wilks’ λ = 0.09, F2_16,272_ = 39.86, P < 0.01). The analysis of covariances (ANCOVA) further showed a significant difference in all head measurements except for head length (Table 3). For their body size, rock-dwelling species had narrower heads than arboreal species and flatter heads than all other habitat groups. Arboreal species have shorter lower jaws, jaw out-levers, and snouts but a longer in-lever for jaw closing than the other two habitat groups.

**Table 2:** Morphology, bite force, and the index of relative importance (IRI) of each dietary item found in the three habitat groups

	Ground			Rock			Arboreal		
	*Females*	*Males*	*Juveniles*	*Females*	*Males*	*Juveniles*	*Females*	*Males*	*Juveniles*
Morphology									
N	6	8	7	26	34	27	19	9	11
SVL (mm)	87.13 (9.85)	85.00 (11.17)	32.82 (4.16)	79.99 (5.85)	86.17 (10.84)	42.85 (10.56)	114.09 (8.89)	126.67 (7.93)	63.16 (24.71)
Head length (mm)	20.24 (2.32)	19.77 (1.62)	10.05 (1.01)	19.46 (1.64)	20.98 (3.30)	11.90 (2.21)	26.17 (1.79)	29.26 (2.03)	16.12 (4.41)
Head width (mm)	17.70 (1.05)	17.87 (1.41)	9.07 (1.01)	16.19 (1.85)	17.41 (2.26)	10.65 (1.85)	21.93 (1.19)	25.71 (1.40)	13.90 (4.30)
Head height (mm)	11.65 (0.76)	11.38 (1.39)	6.07 (0.61)	9.82 (1.38)	10.26 (1.52)	6.80 (0.84)	15.52 (0.86)	18.34 (1.65)	9.56 (2.67)
Lower jaw length (mm)	22.43 (2.79)	22.05 (4.23)	8.61 (0.99)	19.74 (1.99)	21.89 (3.50)	11.29 (3.41)	24.47 (1.73)	28.13 (2.05)	15.06 (4.28)
Jaw out-lever (mm)	19.38 (1.90)	19.25 (2.78)	8.09 (0.94)	17.60 (1.31)	19.35 (2.79)	10.08 (2.58)	22.67 (1.34)	26.27 (2.10)	13.68 (4.51)
Snout Length (mm)	14.92 (2.57)	14.40 (3.76)	5.09 (0.73)	13.74 (2.29)	15.53 (2.69)	6.97 (2.76)	13.13 (1.01)	14.69 (1.37)	7.98 (2.07)
In-lever for jaw opening (mm)	3.06 (1.20)	2.80 (1.51)	0.53 (0.25)	2.14 (1.31)	2.54 (1.52)	1.21 (1.02)	1.79 (0.73)	1.86 (0.46)	1.38 (0.85)
In lever for jaw closing (mm)	4.46 (1.15)	4.85 (1.60)	3.00 (0.51)	3.86 (1.92)	3.82 (1.33)	3.11 (0.50)	9.54 (1.31)	11.58 (1.57)	5.71 (2.51)
Performance									
Bite force (N)	16.11 (3.77)	16.08 (6.25)	1.20 (0.83)	17.77 (8.08)	23.05 (10.19)	4.28 (5.03)	33.83 (7.65)	79.42 (17.09)	10.66 (10.86)
Diet (IRI)									
N		2	6	10	9	19	10	5	6
Ant		92.51 (47.48)	154.77 (27.89)	57.98 (49.97)	69.47 (21.92)	92.59 (29.79)	39.13 (40.07)	55.46 (18.07)	103.33 (32.58)
Hymenoptera		0.12 (0.089)	0.013 (0.032)	2.35 (7.28)	1.25 (1.28)	0.26 (0.34)	0.25 (0.45)	0.33 (0.74)	0.23 (0.25)
Coleoptera		2.82 (1.47)	0.21 (0.47)	7.61 (14.10)	1.76 (1.38)	1.18 (1.61)	0.42 (1.24)	0.46 (1.02)	0.17 (0.11)
Hemiptera			0.037 (0.090)	1.58 (4.85)	0.049 (0.094)	0.077 (0.12)			0.0018 (0.0044)
Diptera			0.020 (0.050)	0.036 (0.10)	0.030 (0.049)	0.016 (0.036)			0.0044 (0.0087)
Diplopoda				0.0014 (0.0035)	0.041 (0.12)	0.013 (0.033)			
Lepidoptera (larvae)				0.77 (2.43)	0.0047 (0.014)	0.0002 (0.0009)			0.0087 (0.021)
Orthoptera				0.03 (0.10)	0.0019 (0.0057)				0.026 (0.065)
Snail									0.0005 (0.012)
Ephemoptera									0.0005 (0.0013)
Isoptera				1.70 (5.37)					
Isopoda						0.0002 (0.001)			

*N* = sample size. IRI values were multiplied by 100 to facilitate the reading of the table

**Table 3:** ANCOVAs performed on head morphological variables testing for differences between habitat groups

*Variable*	*d.f*	*F*	*P*
Head length	2, 143	2.11	0.13
Head width	2, 143	4.74*	0.01
Head height	2, 143	51.53*	< 0.01
Lower jaw length	2, 143	10.44*	< 0.01
Jaw out-lever	2, 143	7.20*	< 0.01
Snout length	2, 143	59.54*	< 0.01
In-lever for jaw opening	2, 143	4.55*	0.01
In-lever for jaw closing	2, 143	54.22*	< 0.01

*Mean difference significance at α < 0.05

An ANOVA testing for differences in absolute bite force between habitat groups was significant (F_2,144_ = 11.99; P < 0.01). Post-hoc tests indicated that ground-dwelling and rock-dwelling species have a lower bite force than arboreal species but did not differ from one another (Table 2). The ANCOVA with lower jaw length and the jaw out-lever as co-variates again detected significant differences in bite force between habitat groups (F_2,142_ = 10.54; P < 0.01). Post-hoc pairwise comparisons on the bite force residuals showed that ground-dwelling species have the lowest relative bite force compared to the other habitat groups.

### Diet

**Fig. 1:**
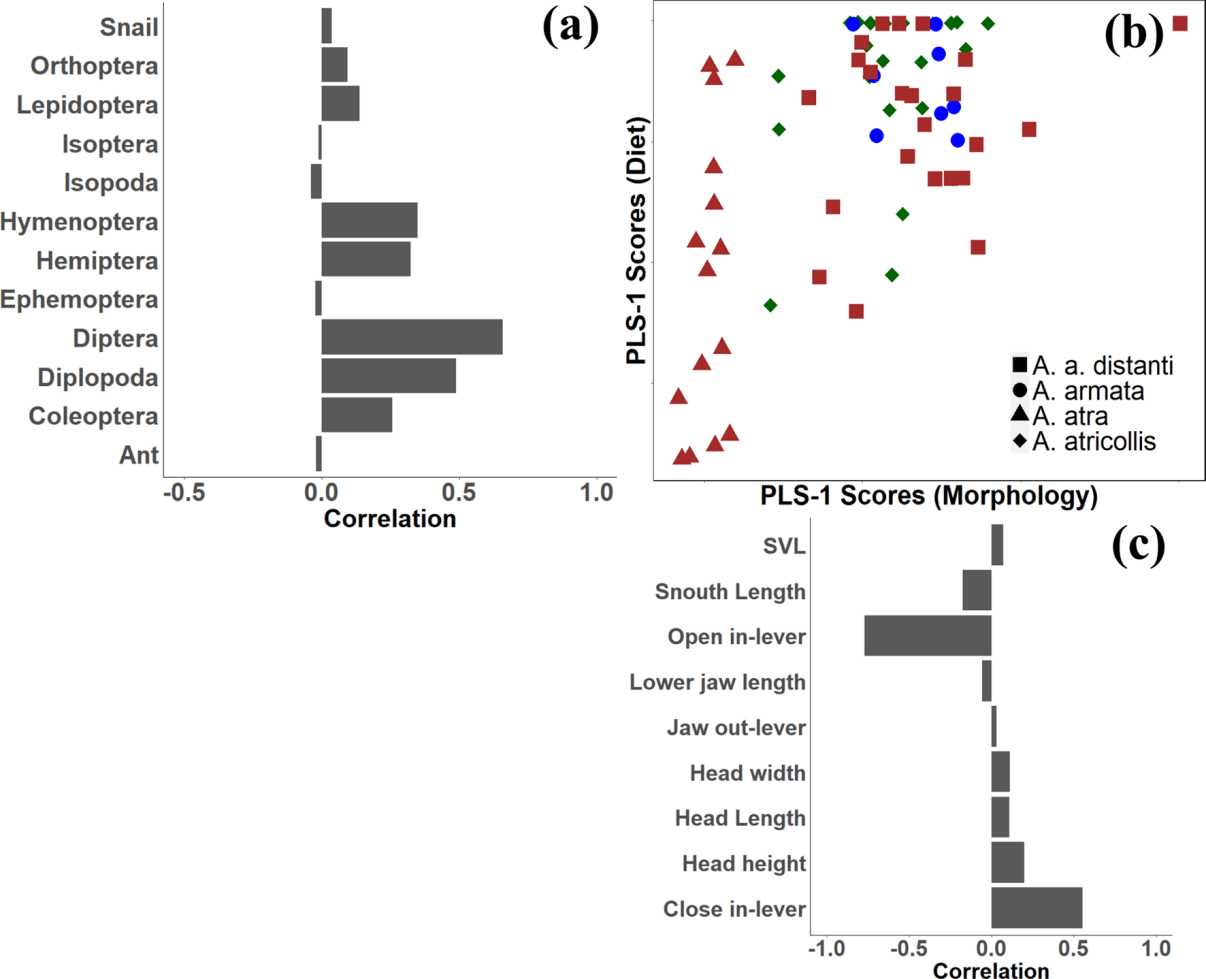
**a** and** c** Bar plots represent the correlations observed between the original variables and the scatterplot axes. **b** Scatterplot of the scores of the four southern African agamid species obtained from partial least-squares (PLS) analysis between absolute head morphological variables (not corrected by size) and indexes of relative importance (IRI) of each prey item. Colour refers to different habitat groups: blue circles, ground dwelling, *Agama armata*; brown triangles and squares, rock dwelling, *A. atra* and *A. a. distanti* respectively; green diamonds, arboreal, *Acanthocercus atricollis*

To understand the relationship between morphology and diet, we ran two-bock partial least-square analyses with snout-vent-length and head measurements versus the prey IRI data. This revealed a significant association between head shape and diet composition (*r* = 0.50, *P* < 0.01). Higher IRI values of Hymenoptera, Diptera, and Diplopoda are associated with a shorter in-lever for jaw opening and longer closing in-lever (Fig. 1). After correcting for size, the significant association remains (*r* = 0.58, *P* < 0.01). The same covariation was observed, suggesting that body and head size are less important drivers of the covariation between diet and morphology. Pearson correlations between bite force and prey IRI indicated a significant negative correlation between absolute bite force and the IRI of ants (*r* = − 0.45, *P* = 0.02) while residual bite force was negatively correlated with the IRI of Hemiptera (*r* = − 0.23, *P* = 0.03), Diptera (*r* = − 0.35, *P* < 0.01), and Diplopoda (*r* = − 0.48, *p* < 0.01).

There was a significant difference in diet between the habitat groups (Wilks’ lambda = 0.50; *F*_24,106_ = 1.82; *P* = 0.02). Univariate ANOVAs identified significant differences in several taxonomic groups (Table 4). Particularly, the relative importance of ants in the diet was highest in ground-dwelling species compared to arboreal species (post-hoc tests). In rock-dwelling species the relative importance of Hemiptera and Diptera in the diet was higher than in arboreal species (Table 2).

**Table 4:** Results of the ANOVAs testing for differences in prey IRI between habitat groups

*Prey IRI*	*d.f*	*F*	*P*
Ants	2, 64	5.15*	< 0.01
Hymenoptera	2, 64	2.13	0.13
Coleoptera	2, 64	0.83	0.44
Hemiptera	2, 64	8.35*	< 0.01
Diptera	2, 64	3.49*	0.04
Diplopoda	2, 64	3.00	0.06
Lepidoptera	2, 64	0.30	0.74
Orthoptera	2, 64	0.21	0.81
Snails	2, 64	1.10	0.34
Ephemoptera	2, 64	1.10	0.34
Isoptera	2, 64	0.37	0.69
Isopoda	2, 64	0.37	0.69

*Mean difference significance at α < 0.05

### Phylogenetic and ontogenetic differences between habitat groups

Phylogenetic ANOVAs identified differences in body size, head variables and bite force between habitat groups (Additional file 2: Table S2). No significant phylogenetic differences were found in diet (although due to the limited observations of certain prey items—F statistics and P-values are unreliable; Additional file 2: Table S3).

Habitat groups differed significantly in body size in adults (F2, 99 = 105.80, P < 0.01) and juveniles (F2, 42 = 12.15, P < 0.01). The multivariate analyses of covariance (MANCOVA) with SVL as covariate showed significant differences in head shape between habitat groups in both adults (Wilks’ λ = 0.08, F16, 182 = 28.17, P < 0.01) and juveniles (Wilks’ λ = 0.06, *F*_16,68_ = 13.11, P < 0.01). The analysis of covariances (ANCOVA) further revealed a significant difference in all head variables except for head length and width for adults and in head height and closing in-lever for juveniles (Additional file 3: Table S4). Habitat groups differed in juveniles (F2, 42 = 7.609, P < 0.01) and adults (F2, 99 = 35.16, P < 0.01) for absolute bite force. Stepwise regression resulted in a single significant model (R^2^ = 0.71; P < 0.01) with the head width (β = − 0.35) and jaw out-lever (β = 1.25) as significant predictors of bite force for adults while the snout length (β = 0.37) was a significant predictor in the model (R^2^ = 0.96; P < 0.01) for juveniles. However, ANCOVA also showed significant differences in bite force between habitat groups in adults (F_2,142_ = 10.54; P < 0.01) and in juveniles (F_2,41_ = 6.16; P < 0.01). We found differences in diet between the habitat groups in both adults (MANOVA; Wilks’ lambda = 0.26, *F*_18,50_ = 2.66, P < 0.01) and juveniles (MANOVA; Wilks’ lambda = 0.14, *F*_22,36_ = 2.77, P < 0.01). Univariate ANOVAs identified significant differences in several prey groups (Additional file 3: Table S5).

## Discussion

Our study shows that the association between external morphology and performance traits can be variable. We expected lizards with longer, wider, and taller heads to bite relatively harder. However, rock-dwelling lizards with narrower and flatter heads showed relatively greater bite force than ground-dwelling lizards. Although ground-dwelling species have taller heads compared to the rock-dwelling relatives, they had the weakest relative bite force. Commensurate with our last prediction, we found evidence of a correlation for lizards with a longer closing in-lever and harder prey in their diet.

Contrary to other ecomorphological studies [5, 6, 11, 17, 29], body and head length were not the best predictors of bite force in our study. Instead, jaw out-lever and lower jaw length (a good proxy for head length [11]) were the best predictors of bite force (see also [30]). This suggests that these simple external head measurements are informative and are valid predictors of bite force despite the complexity of the jaw systems in lizards. These results are counter-intuitive at first, however, as a longer lower jaw should increase the out-lever of the system and hence decreases rather than increase bite force [11]. In our data we find, however, that longer lower jaws and longer jaw out-levers are the best predictors of bite force. Possibly, these two variables stand out as they have low measurement error relative to other head measures which are all very highly positively correlated to bite force (Table 1). Thus, despite the fact that these two variables were retained by the multiple regression the real pattern is one of overall bigger heads being associated with higher bite forces, conforming to our hypothesis that lizards with greater head dimensions bite harder. Approaches such as geometric morphometrics may be particularly suited to better understand whether some parts of the head or cranium are particularly good predictors of bite force in these lizards [4, 31]. Aside from jaw architecture, other factors such as muscle mass, the orientation of the muscle force vectors, fibre length, muscle insertion sites, and the cross sectional area of adductor muscle also participate in the production of bite force and should not be neglected [10, 32].

Arboreal agamid species were the biggest, and had the largest heads and bites forces in absolute terms compared to species from other habitats (Table 2). Bite force show positive allometry relative to body dimensions in many lizards, possibly explaining why the largest species had disproportionately large absolute bite forces [18]. Irrespective of variation in overall size, rock-dwelling species had narrower and flatter heads than lizards from the other habitat groups. This finding suggests that flattened and narrow heads (and body) may confer an advantage to rock-dwelling species and allow them to use rock crevices as shelters to hide from predators [4, 5]. This has been demonstrated in other lizards and the morphological convergence in head shape previously described for rock-dwelling lizards [12, 28] thus appears to be a more general phenomenon. However, flatter heads are typically associated with a weaker bite force [6, 11, 33]. In our study, animals living in rocky habitats bite harder relative to their jaw size and out-lever compared to ground-dwelling species (see also [4, 31]). One possible explanation for this pattern could be differences in non-measured traits such as muscle mass and architecture [7, 30] as well as muscle orientation [20]. For example, a greater jaw adductor muscle mass or changes in muscle architecture (e.g., degree of pennation) can contribute to an increase in absolute and relative bite force [7]. In contrast to rock-dwelling species, arboreal species possess shorter jaws and out-lever lengths but a larger closing in-lever which directly promotes bite force [10, 29]. By reducing the jaw out-lever length and increasing the space available for muscles, bite force is increased [7, 10]. The need for a high bite force could possibly be explained by the hardness of the prey encountered in this environment [9]. A recent study [13], for example, demonstrated that the evolution of cranial shape in Amphibolurines (Agamidae) was significantly associated with their habitat use (arboreal, terrestrial, and rock-dwelling). Major patterns were found in the variation in snout length, skull height, and amount of space for jaw muscles: all of which related to adaptations to bite force generation and prey capture efficiency [13]. Further phylogenetic comparative studies on the relationship between head shape and habitat use are needed to confirm the generality of these results.

Our PLS results suggest that body size is not among the most important drivers of diet. We predicted an association between body size and the range of arthropod orders taken [34]. This was not the case in the agamas included in our study, however. This is consistent with the observation that the arboreal species, *Acanthocercus atricollis*, did not have a wider niche breadth compared to other agama species even though it was the largest species [24]. However, our analyses did demonstrate a significant covariation between head morphology and diet. This demonstrates that the jaw lever system is closely linked to diet, more specifically, the types of prey captured. We found evidence for significant correlation with a longer closing in-lever and a higher relative importance of Hymenoptera and Diplopoda (Fig. 1), both of which are considered to be rather hard prey (see [17]). The fact that head length, width, and height did not show strong covariation with our diet proxies disagrees with observations for other lizards [5, 6, 8, 35]. Given the complexity of the jaw system, a reduced jaw opening and increased jaw closing in-lever can nevertheless enhance bite force [6, 7, 9, 10]. As a result, harder prey can be captured and thus handled more efficiently [18]. However, as to why Diptera, a soft arthropod was positively correlated with in-lever size remains unclear. Larger jaw opening muscles could facilitate faster jaw movements which could allow lizards to specialise on mobile prey such as dipterans, yet the opening in-lever did not co-vary strongly with the relative importance of Diptera in the diet [30].

As expected, the importance of ants in the diet was found to be greatest in ground-dwelling species, while rock-dwelling species seem to consume more dipterans and hemipterans (Table 2). This conforms to differences detected previously [24]. Although only partly significant, both rock species (*Agama aculeata distanti* and *A. atra*) appear to be generalists consuming a more diverse prey spectrum than other agama species, hence having the highest niche breadth [24]. Smaller ground-dwelling species with lower absolute bite force (as a result of their smaller heads) should select softer and smaller prey even if they are not physically constrained to take harder prey [6]. The observed importance of ants in the diet of ground-dwelling species could simply reflect the lack of other prey in the environment, instead of a dietary specialisation. It is possible that agamas do not choose their prey but rather capture whichever arthropod occurs in the environment. Ants are easy to capture and abundant in arid ecosystems [36] and may thus be a profitable prey source. Indeed, the energetic benefit of consuming copious quantity of small hard prey items with minimal search time could outweigh the cost of increased handling time [37]. Future studies on prey availability at these study sites would be of interest for two reasons: (a) to determine the abundance and types of arthropods occurring naturally, thus allowing to understand whether agamas actively select their prey or are opportunistic predators; (b) to reconstruct the original prey size using the arthropods collected as a reference [5].

Phylogenetic analyses revealed no significant association between clade membership and differences found between habitat groups, although our analyses were compromised due to limited number of species (smaller than recommended to conduct phylogenetically informed analyses). Due to low sample size in terms of adults and juveniles (see Additional file 3), the detection of ontogenetic differences might be restricted by low statistical power and hence leads to the inability to make robust inferences.

Our results suggested that variation in head shape cannot be explained by habitat use alone. Selection for head shape and bite force are also highly relevant to territory or predator defence [38]. Sexual selection is likely an important force in driving variation in head morphology in lizards [4]. Differences in head or skull shape between sexes have been demonstrated in many lizards: lacertids [7], *Anolis* lizards [30] and chameleons [8]. However, sexual dimorphism could not be tested in our present study due to the low sample size. Sexual differences in reproductive strategy can translate into differences in bite force given the role of biting behaviours in mating and intrasexual aggression [19, 38]. A previous study has suggested that the larger and wider heads in male *A. atricollis* may be explained by sexual selection [25]. The complex interaction between natural and sexual selection can, however, only be unravelled through comparative studies of the degree of sexual dimorphism in relation to the ecological context of the species based on the quantification of possible variation in diet [4].

## Conclusion

In summary, our data suggests that rock-dwelling southern African agamid species have high relative bite forces despite having narrow and flat heads, suggesting an important role for differences in muscle architecture and/or skull shape in driving these patterns. Moreover, differences in bite force and feeding ecology between habitat groups are not reflected by differences in body and head size (but see [6, 11]). Although niche divergence in diet and habitat use are found to be consistent with variation in cranial morphology and bite force, the potential contribution of sexual selection in driving some of the observed differences needs to be explored.

## Methods

### Study organisms

A total of 147 individuals, including 51 Females, 51 males and 45 juveniles representing six species occupying different habitat types were sampled (see Additional file 1: Table S1). We distinguish male agamas from females visually, based on the bulging of the hemipenes in males [22]. Lizards were caught by hand or noose in different localities in South Africa. *Agama atra* samples (N = 41) were captured in the Muizenberg mountains (34° 05′ S, 18° 26′ E) and the Grootwinterhoek reserve (33° 09′ S, 19° 05′ E) and other parts of the Western Cape in March 2008 and January 2011. *Agama aculeata distanti* (N = 36) were sampled in Kruger National Park (23° 58′ S, 31° 31′ E) and Welgevonden Reserve (24° 12′ S, 27° 54′ E), Limpopo province, in November 2011 and March 2017 respectively. Both *A. anchietae* (N = 10) and *A. aculeata aculeata* (N = 10) were sampled in Tswalu game reserve (27° 17′ S, 22° 23′ E), Northern Cape, in January 2010, with the exception of three *A. anchietae* from Gobabis (22° 26′ S, 18° 57′ E) and Swakopmund (22° 15′ S, 15° 4′ E), Namibia, and one *A. a. aculeata* from Zwartskraal farm (33°10′S, 22°34′E), Western Cape. *Agama armata* (N = 11) were collected at Alicedale Farms (22° 38′ S, 30° 08′ E) and Greater Kuduland Safaris (22° 32′ S, 30° 40′ E) in January 2010 and February 2017. Lastly, *Acanthocercus atricollis* (N = 39) were caught in the suburban area of Mtunzini (28° 57′ S, 31° 44′ E) and Zululand Nurseries, Eshowe (28° 52′ S, 31° 28′ E), KwaZulu-Natal, in February 2017. The GPS coordinates of each lizard were recorded upon capture. Lizards were marked with a non-toxic marker (to avoid recapture), placed in cloth bags and then transferred back to the field station where they were stomach flushed. Morphological and bite force measurements were also taken. Once all the data were collected, we released the animals at the exact location where they were found.

### Morphometrics

**Fig. 2:**
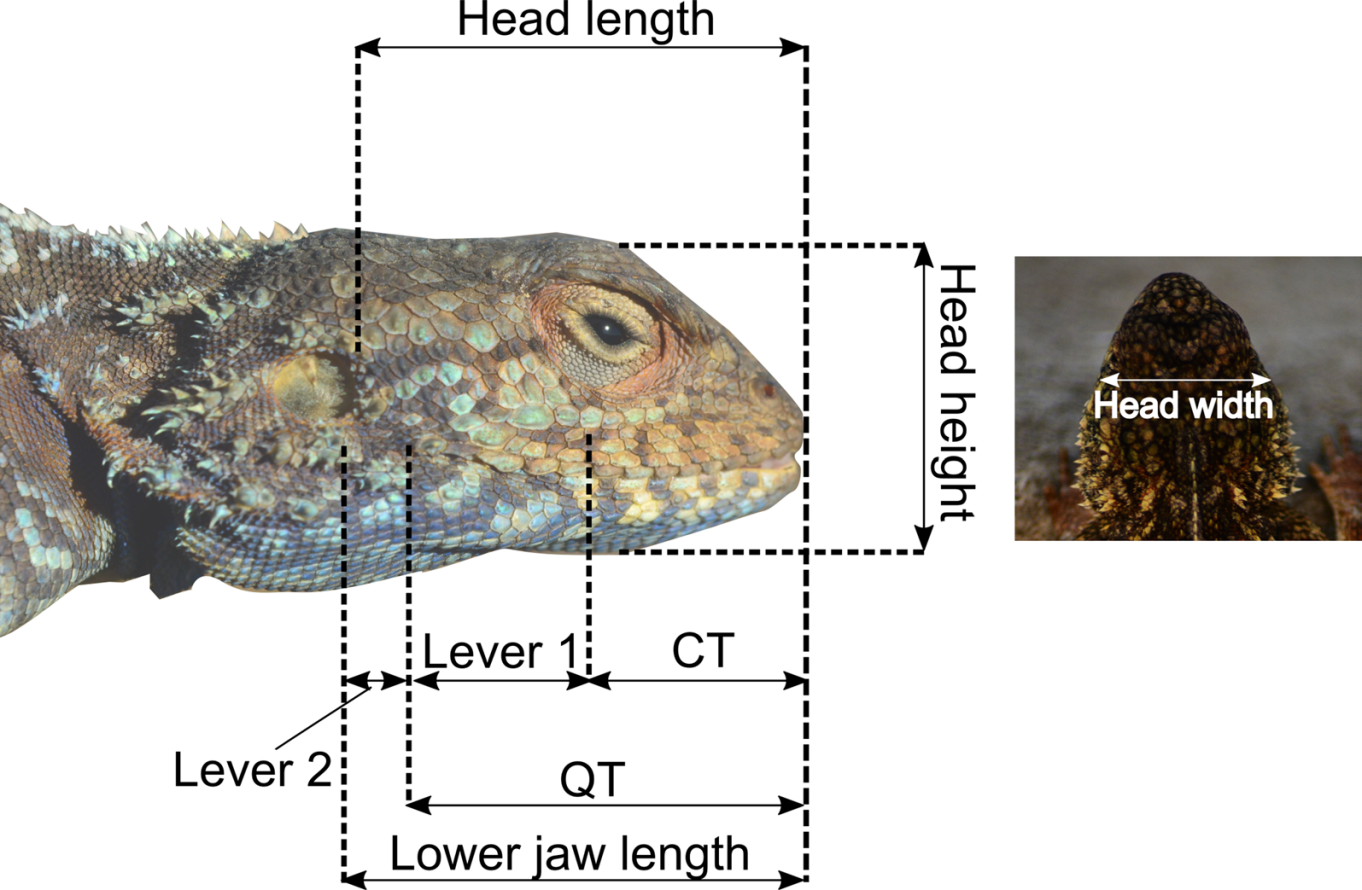
Eight head measurements recorded for each lizard. CT, snout length; QT, quadrate to snout tip, Lever 1, first or closing in-lever; Lever 2, second or opening in-lever

Morphological variables were measured for each individual using digital callipers (Mitutoyo; precision 0.01 mm) according to [11] for the following morphological traits (Fig. 2): snout-vent length (SVL); head width (HW) taken at the widest point of the skull; head length (HL) taken from the tip of the snout to the end of the parietal bone; head depth or height (HH) at the tallest part of the head, posterior to the orbital region; lower jaw length (LJL), taken from the snout tip to the end of the retroarticular process; jaw out-lever taken from the posterior end of the quadrate to snout tip (QT), and the distance from the back of the jugal to the tip of the snout (CT). Based on the latter three measurements, two other morphological variables were calculated: the first or closing in-lever of the jaw being the difference between QT and CT; and the second or opening in-lever, being the subtraction of QT from LJL [9, 17]. A longer in-lever for jaw closing provides a higher mechanical advantage and subsequently increases bite force for a given head size [10].

### Bite force

Bite forces were measured in vivo following the method of Herrel et al. (2001) [6] using an isometric Kistler force transducer (type 9203, 500 N, Kistler Inc. Winterthur, Switzerland), connected to a Kistler charge amplifier (type 5995A) with all measurements made accurate to 0.1 N. A pair of metal bite plates was placed between the jaws of the lizard which typically results in prolonged and repetitive biting. If needed, sides of the jaw were gently tapped to provoke the lizards to bite the plates. Agamids have solid acrodont teeth implanted onto the jaw and are unlikely to suffer any damage from measuring bite force with metal plates. No audible breaking of teeth was present (contra [39]) and inspection of the teeth showed no damage. Our experience with these and other lizards is not consistent with the findings of Lappin & Jones (2014) [39] and suggest that bites on metal plates provide more accurate measures of maximal bite force. The distance between the plates and the point of application of the bite force were standardised across all animals. Bite force was recorded five times for each animal. The maximum value was then retained as the maximal bite force and used in further analyses. Although air temperatures, humidity and other environmental conditions could not be controlled in this study due to the absence of facilities in the field, we ensured that the lizards were tested at the temperatures at which they are active in the field.

### Diet

Data on stomach contents for 67 individuals from four species of agamas (*Agama atra, A. aculeata aculeata, A. armata and Acanthocercus atricollis*) were extracted from a previously published study (Additional file 1: Table S1) [24]. Stomach contents were classified to the lowest taxonomic level possible. The food items were then blotted dry, measured and weighed using an electronic microbalance (AE100-S, Mettler Toledo GmBH, Zurich, Switzerland; ± 0.1 mg) [11].

For each prey group, we calculated the index of relative importance (IRI) [40]. This compound index indicates the importance of particular prey group and provide a balanced view based on combination of unique individual properties (numbers, mass and occurrence in diet) [17, 24]:

$${\text{IRI}} = \left( {\% {\text{N}} + \% {\text{V}}} \right) \times \% {\text{Oc}}$$

where %N is the percentage of numeric abundance, counted from the number of heads of the prey items, %V is the proportion of mass of that prey group to total prey mass and %Oc is frequency of occurrence of a certain prey group.

### Statistical analyses

All morphological and performance variables were logarithmically transformed (log10) before analyses to fulfil the assumptions of normality and homoscedascity. To explore which head variables best explain variation in bite force, stepwise multiple regression analyses were conducted. Pearson correlations were further used to explore relationships between head morphology and bite force.

We grouped the species examined into three habitat groups: rock-dwelling, ground-dwelling, and arboreal. We should point out, however, that these ecological habitat groups may only apply to the populations sampled in our study and are based on our observations in the field. Following the classification of species, we tested whether habitat groups differ in size (SVL) using a univariate analysis of variance (ANOVA). If habitat groups were significantly different in size, multivariate analyses of covariance (MANCOVA) were performed to test for differences in head morphology with SVL as a covariate. We did not test for potential differences between sexes due to the low sample size for each sex per habitat group. Subsequent Tukey’s honest significant difference (HSD) post hoc tests were conducted to test for differences between pairs of habitat groups.

We then investigated whether species assigned to different habitat groups differed in absolute bite force using an ANOVA. A subsequent ANCOVA with the most significant explanatory variables from stepwise regression as covariates was performed to examine whether differences remained when correcting for head dimensions. If so then this would suggest variation in the underlying muscle architecture.

To explore the multivariate association between morphology and diet, we used two-block partial least-squares regressions (PLS) using the two.b.pls function of the geomorph package [41]. Snout vent length and all head variables were computed as the first block of variables while the IRI of the different prey groups were combined in the second block of variables. We first performed the PLS with absolute variables and then repeated the analysis with relative size, in which we used corrected morphological variables using the residuals of each trait following a regression on SVL. We additionally ran a Pearson correlation between absolute bite force and the IRI of all prey and reran the same analysis with size-corrected (residual) bite force. Finally, we tested whether habitat groups differed in their diet composition using a MANOVA and Tukey’s HSD post hoc tests on the IRI of all prey groups.

Using the same statistical analyses above, we tested for ontogenetic differences in the habitat groups. To determine whether differences found between habitat groups are linked to ecological divergence or clade membership, we conducted phylogenetic ANOVAs among the groups using a trimmed phylogeny of Leaché et al. (2014) [21].

All statistical analyses were performed using R v.3.6.2 [42].

## Supplementary Information

**Additional file 1: Table S1.** Morphology, bite force, and the index of relative importance (IRI) of each dietary item found in all six species. N = sample size. IRI values were multiplied by 100 to facilitate the reading of the table. Standard deviation is shown in brackets.

**Additional file 2: Table S2.** Results of phylANOVAs performed on size, head variables and bite force testing for differences between habitat groups due to phylogenetic relationship. The averages of each species were taken for the calculation of each variable. **Table S3.** Results of phylANOVAs performed on prey IRI testing for diet differences between habitat groups due to phylogenetic relationship.

**Additional file 3: Table S4.** ANCOVAs performed on head morphological variables testing for differences in adults (N for ground dwelling = 14, rock dwelling = 16, arboreal = 28) and juveniles (N for ground dwelling = 7, rock dwelling = 27, arboreal = 11) among habitat groups. **Table S5.** Results of the ANOVAs testing for differences in prey IRI in adults (N for ground dwelling = 2, rock dwelling = 19, arboreal = 15) and juveniles (N for ground dwelling = 6, rock dwelling = 19, arboreal = 6) between habitat groups.

## Data Availability

The datasets used and/or analysed during the current study are available in Zenodo: https://doi.org/10.5281/zenodo.4581097
