# Supplementary material for: The relationship between bite force, morphology, and diet in southern African agamids
Source: BMC Ecol Evol. 2021 Jun 21;21:126. doi: 10.1186/s12862-021-01859-w (PMC8215774; doi:10.1186/s12862-021-01859-w)
Supplement: Supplementary file 1 — Additional file 1: Table S1. Morphology, bite force, and the index of relative importance (IRI) of each dietary item found in all six species. N = sample size. IRI values were multiplied by 100 to facilitate the reading of the table. Standard deviation is shown in brackets. [file 12862_2021_1859_MOESM1_ESM.docx]

**Table S1. Morphology, bite force, and the index of relative importance (IRI) of each dietary item found in all six species. *N* = sample size. IRI values were multiplied by 100 to facilitate the reading of the table. Standard deviation is shown in brackets.**

|  | ***Agama aculeata aculeata*** | | |  | ***Agama aculeata distanti*** | | | | |  | ***Agama anchietae*** | | |
| --- | --- | --- | --- | --- | --- | --- | --- | --- | --- | --- | --- | --- | --- |
|  | Females |  | Males |  | Females |  | Males |  | Juveniles |  | Females |  | Males |
| *Morphology* |  |  |  |  |  |  |  |  |  |  |  |  |  |
| ***N*** | 5 |  | 5 |  | 9 |  | 6 |  | 21 |  | 1 |  | 9 |
| **SVL (mm)** | 87.94 (10.78) |  | 91.93 (7.27) |  | 80.73 (4.93) |  | 74.50 (5.50) |  | 38.82 (5.26) |  | 86.98 (NA) |  | 89.85 (12.17) |
| **Head length (mm)** | 20.47 (2.51) |  | 20.64 (1.37) |  | 19.16 (0.82) |  | 18.74 (1.02) |  | 10.93 (1.07) |  | 19.07 (NA) |  | 20.33 (3.43) |
| **Head width (mm)** | 17.70 (1.17) |  | 18.59 (1.27) |  | 17.81 (0.89) |  | 16.97 (0.41) |  | 10.03 (1.58) |  | 16.11 (NA) |  | 18.25 (3.11) |
| **Head hight (mm)** | 11.84 (0.67) |  | 11.59 (1.78) |  | 10.99 (0.81) |  | 10.93 (0.46) |  | 6.57 (0.80) |  | 8.40 (NA) |  | 10.54 (2.23) |
| **Lower jaw length (mm)** | 22.45 (3.12) |  | 24.05 (3.58) |  | 18.49 (1.53) |  | 18.06 (1.47) |  | 9.62 (1.21) |  | 17.79 (NA) |  | 22.37 (4.04) |
| **Jaw out-lever (mm)** | 19.45 (2.12) |  | 20.66 (2.37) |  | 17.30 (1.01) |  | 16.75 (1.11) |  | 8.86 (1.10) |  | 17.27 (NA) |  | 19.56 (3.23) |
| **Snout Length (mm)** | 15.10 (2.83) |  | 16.77 (1.60) |  | 11.47 (2.26) |  | 11.77 (2.41) |  | 5.59 (0.79) |  | 15.72 (NA) |  | 16.37 (2.19) |
| **In-lever for jaw opening (mm)** | 3.00 (1.33) |  | 3.39 (1.22) |  | 1.19 (0.80) |  | 1.31 (0.52) |  | 0.76 (0.34) |  | 0.52 (NA) |  | 2.81 (1.78) |
| **In lever for jaw closing (mm)** | 4.35 (1.26) |  | 3.89 (0.91) |  | 5.83 (1.87) |  | 4.97 (1.37) |  | 3.27 (0.43) |  | 1.55 (NA) |  | 3.19 (1.54) |
| *Performance* |  |  |  |  |  |  |  |  |  |  |  |  |  |
| **Bite force (N)** | 16.26 (4.20) |  | 18.63 (6.37) |  | 11.17 (5.14) |  | 12.63 (5.43) |  | 1.87 (0.85) |  | 17.08 (NA) |  | 18.90 (10.12) |
| *Diet (IRI)* |  |  |  |  |  |  |  |  |  |  |  |  |  |
| ***N*** |  |  |  |  | 5 |  | 4 |  | 14 |  |  |  |  |
| **Ant** |  |  |  |  | 5865.28 (4316.11) | | 7779.80 (954.89) | | 8046.01 (2389.60) | |  |  |  |
| **Hymenoptera** |  |  |  |  | 5.69 (8.07) |  | 39.66 (37.52) |  | 26.95 (38.52) |  |  |  |  |
| **Coleoptera** |  |  |  |  | 117.31 (147.68) | | 110.16 (70.91) | | 117.80 (179.37) | |  |  |  |
| **Hemiptera** |  |  |  |  | 3.02 (3.16) |  | 4.09 (4.77) |  | 7.86 (13.76) |  |  |  |  |
| **Diptera** |  |  |  |  | 6.43 (14.38) |  | 1.37 (1.70) |  | 1.62 (4.19) |  |  |  |  |
| **Diplopoda** |  |  |  |  |  |  |  |  |  |  |  |  |  |
| **Lepidoptera** |  |  |  |  |  |  |  |  |  |  |  |  |  |
| **Orthoptera** |  |  |  |  |  |  |  |  |  |  |  |  |  |
| **Snail** |  |  |  |  |  |  |  |  |  |  |  |  |  |
| **Ephemoptera** |  |  |  |  |  |  |  |  |  |  |  |  |  |
| **Isoptera** |  |  |  |  | 339.65 (759.47) | |  |  |  |  |  |  |  |
| **Isopoda** |  |  |  |  |  |  |  |  | 0.03 (0.11) |  |  |  |  |

**Table S5.** (continued)

|  | ***Agama armata*** | | | | | ***Agama atra*** | | | | | | | ***Acanthocercus atricollis*** | | | | |
| --- | --- | --- | --- | --- | --- | --- | --- | --- | --- | --- | --- | --- | --- | --- | --- | --- | --- |
|  | Females |  | Males |  | Juveniles |  | Females |  | Males |  | Juveniles |  | Females |  | Males |  | Juveniles |
| *Morphology* |  |  |  |  |  |  |  |  |  |  |  |  |  |  |  |  |  |
| ***N*** | 1 |  | 3 |  | 7 |  | 16 |  | 19 |  | 6 |  | 19 |  | 9 |  | 11 |
| **SVL (mm)** | 83.08 (NA) |  | 73.47 (3.38) |  | 32.82 (4.16) |  | 79.14 (6.30) |  | 88.12 (9.22) |  | 56.96 (12.75) | | 114.09 (8.89) | | 126.67 (7.93) | | 63.16 (24.71) |
| **Head length (mm)** | 19.10 (NA) |  | 18.30 (0.53) |  | 10.05 (1.01) |  | 19.65 (2.01) |  | 22.00 (3.38) |  | 15.29 (1.78) |  | 26.17 (1.79) |  | 29.26 (2.03) |  | 16.12 (4.41) |
| **Head width (mm)** | 17.70 (NA) |  | 16.67 (0.56) |  | 9.07 (1.01) |  | 15.28 (1.68) |  | 17.15 (2.14) |  | 12.80 (0.77) |  | 21.93 (1.19) |  | 25.71 (1.40) |  | 13.90 (4.30) |
| **Head hight (mm)** | 10.70 (NA) |  | 11.03 (0.38) |  | 6.07 (0.61) |  | 9.25 (1.23) |  | 9.91 (1.28) |  | 7.63 (0.22) |  | 15.52 (0.86) |  | 18.34 (1.65) |  | 9.56 (2.67) |
| **Lower jaw length (mm)** | 22.32 (NA) |  | 18.71 (3.22) |  | 8.61 (0.99) |  | 20.57 (1.86) |  | 22.87 (2.94) |  | 17.11 (1.57) |  | 24.47 (1.73) |  | 28.13 (2.05) |  | 15.06 (4.28) |
| **Jaw out-lever (mm)** | 19.01 (NA) |  | 16.90 (1.61) |  | 8.09 (0.94) |  | 17.79 (1.50) |  | 20.07 (2.55) |  | 14.31 (1.38) |  | 22.67 (1.34) |  | 26.27 (2.10) |  | 13.68 (4.51) |
| **Snout Length (mm)** | 14.04 (NA) |  | 10.44 (2.63) |  | 5.09 (0.73) |  | 14.90 (1.12) |  | 16.32 (1.94) |  | 11.79 (1.15) |  | 13.13 (1.01) |  | 14.69 (1.37) |  | 7.98 (2.07) |
| **In-lever for jaw opening (mm)** | 3.31 (NA) |  | 1.81 (1.62) |  | 0.53 (0.25) |  | 2.78 (1.16) |  | 2.80 (1.47) |  | 2.80 (1.05) |  | 1.79 (0.73) |  | 1.86 (0.46) |  | 1.38 (0.85) |
| **In lever for jaw closing (mm)** | 4.97 (NA) |  | 6.46 (1.03) |  | 3.00 (0.51) |  | 2.89 (0.77) |  | 3.75 (1.02) |  | 2.53 (0.27) |  | 9.54 (1.31) |  | 11.58 (1.57) |  | 5.71 (2.51) |
| *Performance* |  |  |  |  |  |  |  |  |  |  |  |  |  |  |  |  |  |
| **Bite force (N)** | 15.38 (NA) |  | 11.83 (3.50) |  | 1.20 (0.83) |  | 21.53 (7.32) |  | 28.30 (7.92) |  | 12.71 (4.36) |  | 33.83 (7.65) |  | 79.42 (17.09) | | 10.66 (10.86) |
| *Diet (IRI)* |  |  |  |  |  |  |  |  |  |  |  |  |  |  |  |  |  |
| ***N*** |  |  | 2 |  | 6 |  | 6 |  | 5 |  | 5 |  | 10 |  | 5 |  | 6 |
| **Ant** |  |  | 9251.48 (4748.32) | | 15477.26 (2789.22) | | 15477.26 (2789.22) | | 6281.11 (2770.34) | | 12656.46 (1344.59) | | 3913.39 (4006.96) | | 5545.54 (1806.54) | | 10332.92 (3257.74) |
| **Hymenoptera** |  |  | 11.64 (0.89) |  | 1.29 (3.17) |  | 1.29 (3.17) |  | 193.65 (135.87) | | 22.58 (19.67) | | 24.54 (44.92) | | 32.95 (73.68) | | 22.72 (24.92) |
| **Coleoptera** |  |  | 282.19 (146.54) | | 21.44 (47.47) | | 21.44 (47.47) |  | 228.02 (163.92) | | 118.02 (109.64) | | 42.50 (124.14) | | 45.53 (101.81) | | 17.47 (10.61) |
| **Hemiptera** |  |  |  |  | 3.68 (9.01) |  | 3.68 (9.01) |  | 5.61 (12.54) |  | 7.31 (6.10) |  |  |  |  |  | 0.18 (0.44) |
| **Diptera** |  |  |  |  | 2.03 (4.97) |  | 2.03 (4.97) |  | 4.32 (6.40) |  | 1.63 (1.27) |  |  |  |  |  | 0.44 (0.87) |
| **Diplopoda** |  |  |  |  |  |  |  |  | 7.45 (16.66) |  | 4.90 (5.22) |  |  |  |  |  |  |
| **Lepidoptera** |  |  |  |  |  |  |  |  | 0.85 (1.91) |  | 0.08 (0.17) |  |  |  |  |  | 0.87 (2.14) |
| **Orthoptera** |  |  |  |  |  |  |  |  | 0.34 (0.76) |  |  |  |  |  |  |  | 2.63 (6.45) |
| **Snail** |  |  |  |  |  |  |  |  |  |  |  |  |  |  |  |  | 0.50 (1.22) |
| **Ephemoptera** |  |  |  |  |  |  |  |  |  |  |  |  |  |  |  |  | 0.05 (0.13) |
| **Isoptera** |  |  |  |  |  |  |  |  |  |  |  |  |  |  |  |  |  |
| **Isopoda** |  |  |  |  |  |  |  |  |  |  |  |  |  |  |  |  |  |
