# Supplementary material for: The relationship between bite force, morphology, and diet in southern African agamids
Source: BMC Ecol Evol. 2021 Jun 21;21:126. doi: 10.1186/s12862-021-01859-w (PMC8215774; doi:10.1186/s12862-021-01859-w)
Supplement: Supplementary file 2 — Additional file 2: Table S2. Results of phylANOVAs performed on size, head variables and bite force testing for differences between habitat groups due to phylogenetic relationship. The averages of each species were taken for the calculation of each variable. Table S3. Results of phylANOVAs performed on prey IRI testing for diet differences between habitat groups due to phylogenetic relationship. [file 12862_2021_1859_MOESM2_ESM.docx]

**Table S2. Results of phylANOVAs performed on size, head variables and bite force testing for differences between habitat groups due to phylogenetic relationship. The averages of each species were taken for the calculation of each variable.**

| ***Variable*** | ***d.f.*** | ***F*** | ***P*** |
| --- | --- | --- | --- |
| Snout vent length | 2, 2 | 8.37 | 0.43 |
| Head length | 2, 2 | 14.48 | 0.31 |
| Head width | 2, 2 | 6.66 | 0.48 |
| Head height | 2, 2 | 11.93 | 0.32 |
| Lower jaw length | 2, 2 | 13.48 | 0.31 |
| Jaw out-lever | 2, 2 | 12.78 | 0.33 |
| Snout length | 2, 2 | 12.55 | 0.32 |
| In-lever for jaw opening | 2, 2 | 9.84 | 0.39 |
| In-lever for jaw closing | 2, 2 | 56.20 | 0.10 |
| Bite force | 2, 2 | 13.81 | 0.30 |
|  |  |  |  |
| ** mean difference significance at α<0.05* | | | |

**Table S3. Results of phylANOVAs performed on prey IRI testing for diet differences between habitat groups due to phylogenetic relationship.**

| ***Prey IRI*** | ***d.f.*** | ***F*** | ***P*** |
| --- | --- | --- | --- |
| Ants | 1, 1 | 0.21 | 0.88 |
| Hymenoptera | 1, 1 | 39.27 | 0.26 |
| Coleoptera | 1, 1 | 2.31 | 0.67 |
| Hemiptera | 1, 1 | 1.86 | 0.69 |
| Diptera | 1, 1 | 50.21 | 0.23 |
| Diplopoda | 1, 1 | 1.423x10^32^ | <0.01 |
| Lepidoptera | 1, 1 | Inf | <0.01 |
| Orthoptera | 1, 1 | 1.5018x10^32^ | <0.01 |
| Snails | 1, 1 | 0.00 | 0.00 |
| Ephemoptera | 1, 1 | 0.00 | 0.00 |
| Isoptera | 1, 1 | 0.33 | 0.86 |
| Isopoda | 1, 1 | 0.33 | 0.86 |
|  |  |  |  |
| ** mean difference significance at α<0.05* | | | |
