# Supplementary material for: The relationship between bite force, morphology, and diet in southern African agamids
Source: BMC Ecol Evol. 2021 Jun 21;21:126. doi: 10.1186/s12862-021-01859-w (PMC8215774; doi:10.1186/s12862-021-01859-w)
Supplement: Supplementary file 3 — Additional file 3: Table S4. ANCOVAs performed on head morphological variables testing for differences in adults (N for ground dwelling = 14, rock dwelling = 16, arboreal = 28) and juveniles (N for ground dwelling = 7, rock dwelling = 27, arboreal = 11) among habitat groups. Table S5. Results of the ANOVAs testing for differences in prey IRI in adults (N for ground dwelling = 2, rock dwelling = 19, arboreal = 15) and juveniles (N for ground dwelling = 6, rock dwelling = 19, arboreal = 6) between habitat groups. [file 12862_2021_1859_MOESM3_ESM.docx]

**Table S3. ANCOVAs performed on head morphological variables testing for differences in adults (N for ground dwelling = 14, rock dwelling = 16, arboreal = 28) and juveniles (****N for ground dwelling = 7, rock dwelling = 27, arboreal = 11) among habitat groups.**

|  |  | Adults |  |  |  | Juveniles |  |
| --- | --- | --- | --- | --- | --- | --- | --- |
|  | ***d.f.*** | ***F*** | ***P*** |  | ***d.f.*** | ***F*** | ***P*** |
| Head length | 2, 98 | 1.41 | 0.25 |  | 2, 41 | 1.63 | 0.21 |
| Head width | 2, 98 | 1.93 | 0.15 |  | 2, 41 | 0.42 | 0.66 |
| Head height | 2, 98 | 19.95 | <0.01 |  | 2, 41 | 6.42 | <0.01 |
| Lower jaw length | 2, 98 | 12.9 | <0.01 |  | 2, 41 | 0.09 | 0.91 |
| Jaw out-lever | 2, 98 | 5.55 | <0.01 |  | 2, 41 | 0.15 | 0.86 |
| Snout length | 2, 98 | 27.82 | <0.01 |  | 2, 41 | 2.41 | 0.10 |
| In-lever for jaw opening | 2, 98 | 7.18 | <0.01 |  | 2, 41 | 0.96 | 0.39 |
| In-lever for jaw closing | 2, 98 | 14.56 | <0.01 |  | 2, 41 | 8.38 | <0.01 |
|  |  |  |  |  |  |  |  |
| ** mean difference significance at α<0.05* | | |  |  |  |  |  |

**Table S4. Results of the ANOVAs testing for differences in prey IRI in adults (N for ground dwelling = 2, rock dwelling = 19, arboreal = 15) and juveniles (N for ground dwelling = 6, rock dwelling = 19, arboreal = 6) between habitat groups.**

|  |  | Adults |  |  |  | Juveniles |  |
| --- | --- | --- | --- | --- | --- | --- | --- |
|  | ***d.f.*** | ***F*** | ***P*** |  | ***d.f.*** | ***F*** | ***P*** |
| Ants | 2, 33 | 1.31 | 0.28 |  | 2, 28 | 6.23 | <0.01 |
| Hymenoptera | 2, 33 | 5.85 | <0.01 |  | 2, 28 | 1.76 | 0.19 |
| Coleoptera | 2, 33 | 6.45 | <0.01 |  | 2, 28 | 6.97 | <0.01 |
| Hemiptera | 2, 33 | 7.55 | <0.01 |  | 2, 28 | 1.77 | 0.19 |
| Diptera | 2, 33 | 6.76 | <0.01 |  | 2, 28 | 0.43 | 0.66 |
| Diplopoda | 2, 33 | 1.37 | 0.27 |  | 2, 28 | 1.42 | 0.26 |
| Lepidoptera | 2, 33 | 0.71 | 0.50 |  | 2, 28 | 0.46 | 0.63 |
| Orthoptera | 2, 33 | 0.88 | 0.43 |  | 2, 28 | 2.26 | 0.12 |
| Snails |  |  |  |  | 2, 28 | 2.26 | 0.12 |
| Ephemoptera |  |  |  |  | 2, 28 | 2.26 | 0.12 |
| Isoptera | 2, 33 | 0.43 | 0.65 |  |  |  |  |
| Isopoda |  |  |  |  | 2, 28 | 0.3 | 0.74 |
|  |  |  |  |  |  |  |  |
| ** mean difference significance at α<0.05* | | | |  |  |  |  |
